# Very natural cancer chemoprevention: A research profile of Li-Shu Wang

**DOI:** 10.1002/fft2.35

**Published:** 2020-08-19

**Authors:** Li-Shu Wang

**Affiliations:** Division of Hematology and Oncology, Medical College of Wisconsin, Milwaukee, Wisconsin

Dr. Li-Shu Wang, Associate Professor of Medicine, Division of Hematology and Oncology, Medical College of Wisconsin, was trained in the fields of nutritional biochemistry, cancer biology, carcinogenesis, and chemoprevention using natural products.

Li-Shu obtained her Master of Animal Husbandry from the National Chung-Hsing University, Taichung City, Taiwan in 1999. During this training, she became aware of the ability of many food constituents to prevent human disease, including cancer, and thus decided to further pursue this area of research activity at Ohio State University in the United States. She joined the laboratory of Dr. Young Lin of the College of Veterinary Medicine in 2002 for her Ph.D. thesis training and, in his laboratory, investigated the mechanisms by which conjugated linoleic acid from milk prevents mammary cancer. During her training in Dr. Lin’s laboratory, she developed many of the necessary skills to investigate the mechanisms of cancer prevention by conjugated linoleic acid on a molecular and cellular level. Li-Shu’s research was highly productive and she received independent research funding from the IAMS Company in support of her research. When she completed her Ph.D. thesis, she joined Dr. Gary Stoner’s laboratory and continue in her efforts to prevent cancer with natural food substances.

Since joining Dr. Stoner’s laboratory, Li-Shu worked on projects entitled “Chemoprevention of esophageal cancer with berries” funded by NIH and “Chemoprevention of G.I. tract cancer” funded by United States Department of Agriculture. Li-Shu investigated the mechanisms by which freeze-dried black raspberries (BRBs) prevent cancer in the esophagus and colon. Using a rodent model of esophageal cancer, she has shown that berries modulate the expression levels of multiple cancer genes associated with cellular processes such as proliferation, apoptosis, angiogenesis, inflammation, cell–cell communication, cell signaling pathways, and oxidative processes. This modulation of cancer genes leads to a significant reduction in the development of esophagus cancer in these animals. She also identified the active constituents in berries that are responsible for most, if not all, of their cancer-inhibitory effects, namely the anthocyanins and ellagitannins. Perhaps more importantly, she showed that the short-term treatment of human colon cancer patients with a slurry of berry powder administered orally on a daily basis leads to reduced growth of colon cancers. These effects are due, at least in part, to the inhibitory effects of berries on downregulation of colon cancer genes. Even more exciting, Li-Shu reported that the oral administration of berry powder to patients with a disease called familial adenomatous polyposis leads to a 50% regression rate of rectal polyps. This response is superior to responses that have been reported for nonsteroidal anti-inflammatory drugs, and unlike these drugs, the berries do not appear to have any toxic side effects. Further, Li-Shu showed that the inhibitory effects of the berries are due, at least in part, to their effects on the Wnt pathway, a major signaling pathway in colon cancer. While Li-Shu was at Dr. Stoner’s laboratory as a research scientist, she successfully obtained a research grant entitled “Mechanisms of tumor suppressor gene reactivation in colon cancer by berries” funded by NIH and because of that award and her high productivity, Li-Shu was recruited as an independent faculty member at Medical College of Wisconsin.

After becoming a faculty member at Medical College of Wisconsin, Li-Shu continued to investigate the chemopreventive mechanisms of BRBs where she received another award from American Cancer Society entitled “Mechanisms of anti-inflammation in ulcerative colitis by berries.” Her laboratory had evidence that BRBs cause demethylation and reactivation of tumor suppressor genes in rodent colon and human colon leading to their enhanced expression. The protective effects of BRBs against human and mouse colorectal cancers are associated, at least in part, with their hypomethylation activities. Loss of responses to BRB treatment in humans may be due to decreased sensitivity to BRB-induced DNA hypomethylation. Investigating deeper in the mechanisms of reactivation of tumor suppressor genes by BRBs, studies from Li-Shu’s laboratory suggest that fibers are chemopreventive partly because they can be metabolized by gut microbiota into short chain fatty acids, for example, butyrate. Short-chain fatty acids then activate free fatty acid receptor 2 on the cell membrane and function as histone deacetylase inhibitor in colon epithelium and immune cells such as neutrophils in mice bearing colon tumors. Histone deacetylase, an epigenetic event same as DNA methylation, also controls gene expression. Interestingly, Li-Shu’s lab showed that loss of free fatty acid receptor 2 dampens BRBs’ anti-colon cancer ability in mice. Further, natural killer cells are an essential component of innate immunity against cancer development. Li-Shu’s lab later reported that gut metabolites of BRBs enhance natural killer cell infiltration into the colon that contributes to the suppression of colorectal cancer. These results led to a successfully funding of a project entitled “Enhancing NK cell functions by berry metabolites for colon cancer” funded by United States Department of Agriculture. Because many other commonly consumed plant-based foods can be metabolized by gut microbiota and form metabolites that are very similar and/or same as those of BRB metabolites, results from Li-Shu’s lab could well apply to those foods.

Li-Shu’s observations have major importance in the field of cancer chemoprevention. First, they demonstrate that it is possible to prevent cancer in animals and in humans using a food-based approach. The freeze-drying of berries leads to a 10-fold concentration of their inhibitory components and this is strong enough to lead to cancer preventative effects. It is entirely possible that other foodstuffs would be disease preventative if their inhibitory components were similarly concentrated; thus, this approach could have major significance for the agricultural industry. Second, Li-Shu has shown that berry components and their metabolites modulate the expression of cancer genes involved in many different cellular functions. By restoring the expression of these genes to normal levels, the net effect is to prevent cancer. Finally, the use of the food-based approach to cancer prevention may well be a “safer” alternative to the use of drugs. The recent experience with Vioxx ®, which caused cardiovascular effects leading to death in some patients receiving this agent for prevention of colorectal cancer, underscores the need to develop cancer preventative agents which elicit little or no toxicity. Thus, with her research experience in the rapidly growing field of natural substances in cancer prevention, and her ability to examine the effects of these substances at a cellular and molecular level, Li-Shu has become a major investigator and important player in the field. There is no question that the field of cancer chemoprevention has become a worldwide research priority with increased funding from government and private agencies. Li-Shu stays persistent in her research goal—translate positive findings in in vitro and animal models to humans at high risk for cancer.

## Key Findings

- It is possible to prevent cancer in animals and in humans using a food-based approach.
- Berry components and their metabolites modulate the expression of cancer genes involved in many different cellular functions.
- Finding with berries could well apply to many other commonly consumed plant-based foods because similar/same metabolites are produced when their components are metabolized by gut microbiota.

